# Supplementary material for: Changing the Paradigm-Controlling Polymer Morphology during 3D Printing Defines Properties
Source: Polymers (Basel). 2022 Apr 19;14(9):1638. doi: 10.3390/polym14091638 (PMC9101014; doi:10.3390/polym14091638)
Supplement: Supplementary file 1 [file polymers-14-01638-s001.zip › polymers-1639195-supplementary.pdf]

Supplementary Materials

Changing the Paradigm-Controlling Polymer Morphology during 3D Printing Defines Properties

Daniel P. da Silva[a], João Pinheiro[a], Saba Abdulghani[a], Christina Kamma Lorger[b], Juan Carlos Martinez[b], Eduardo Solano, Artur Mateus[a], Paula Pascoal-Faria[a] and Geoffrey R. Mitchell[a*]

Figures S1 to S4 show the components used in the 3D printer which was designed to be mounted on the ALBA NCD-SWEET beamline and used in this work. The complete printer is shown in Figure 6.

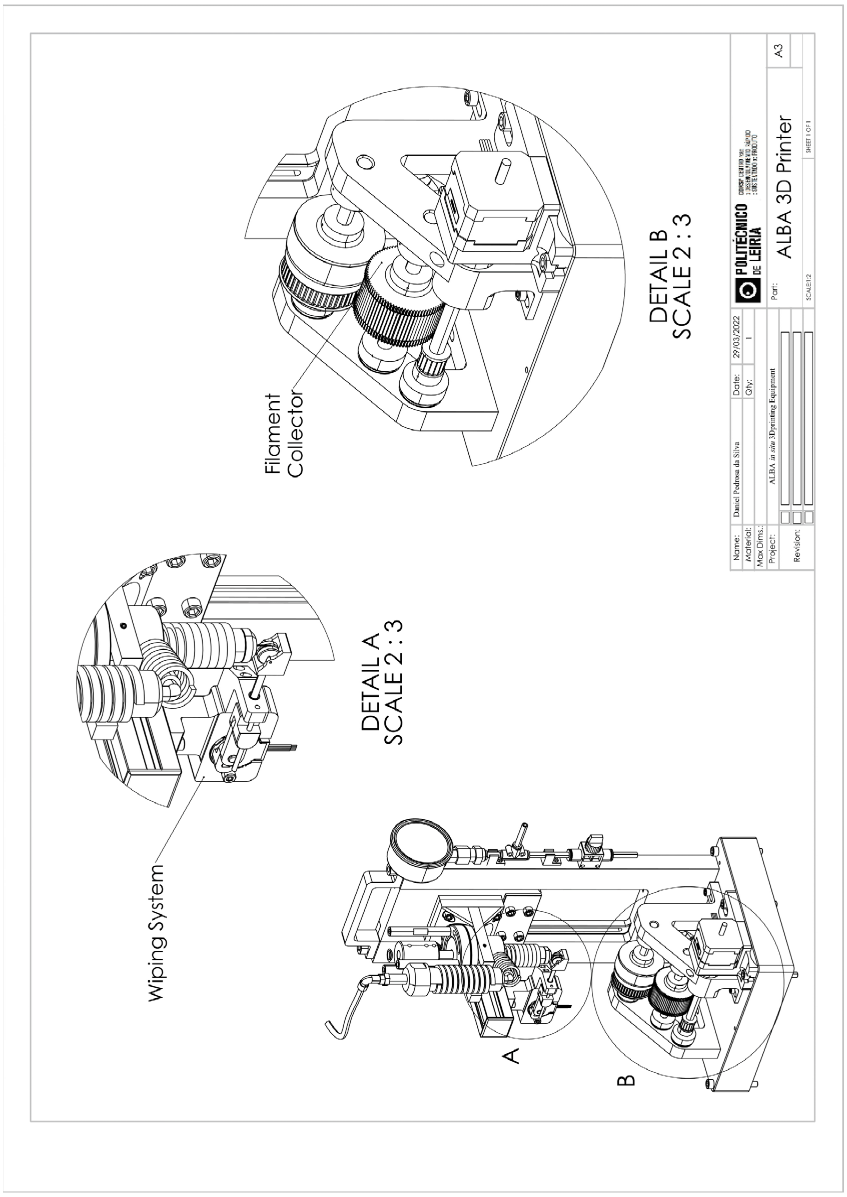

Figure S1—Schematic of the equipment with the collector and winding systems highlighted.

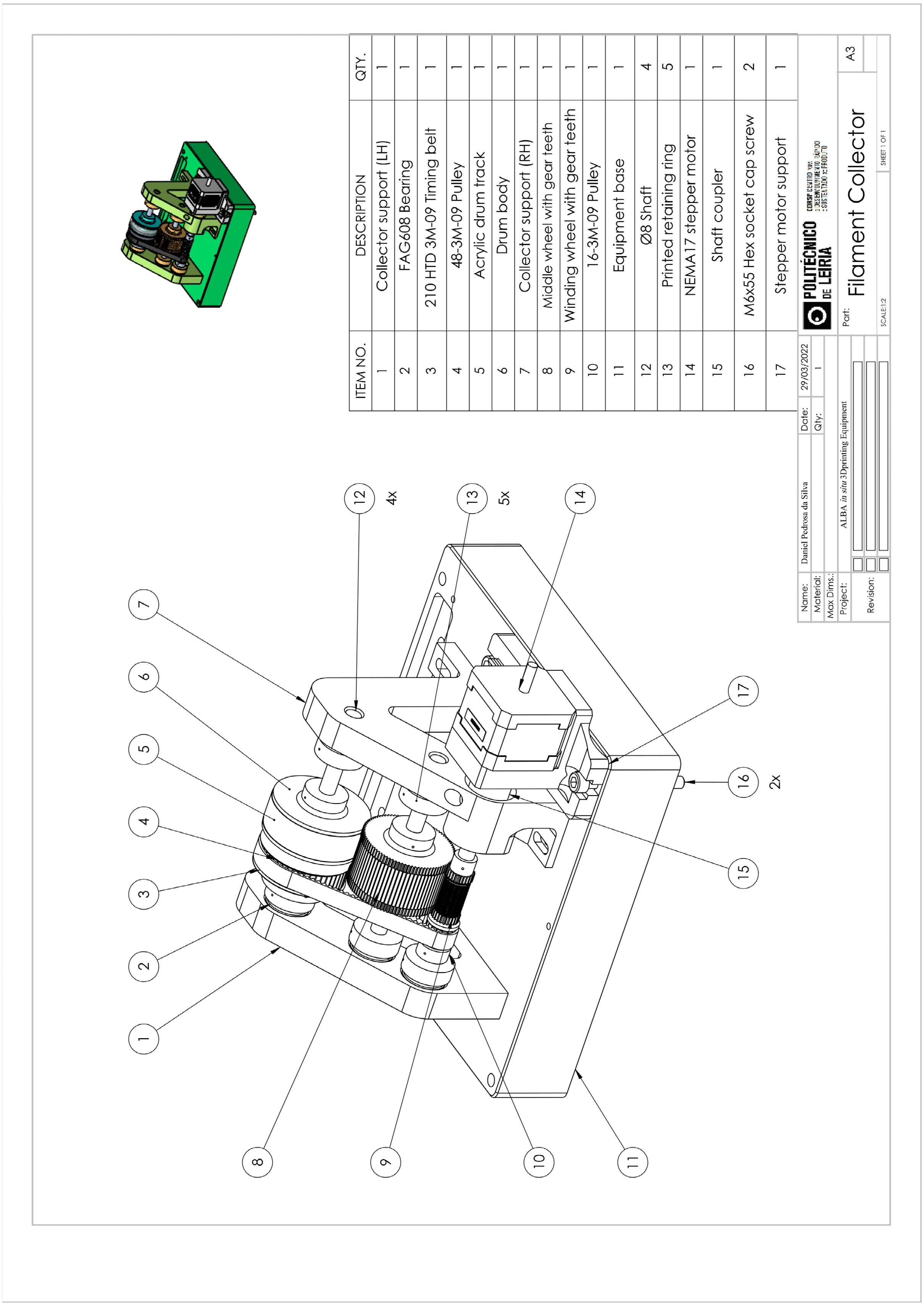

Figure S2—Filament collector components.

Figures S3 and S4 show detailed drawings of the components of the extruder (and feeder) in the 3d printer used at ALBA in this work.

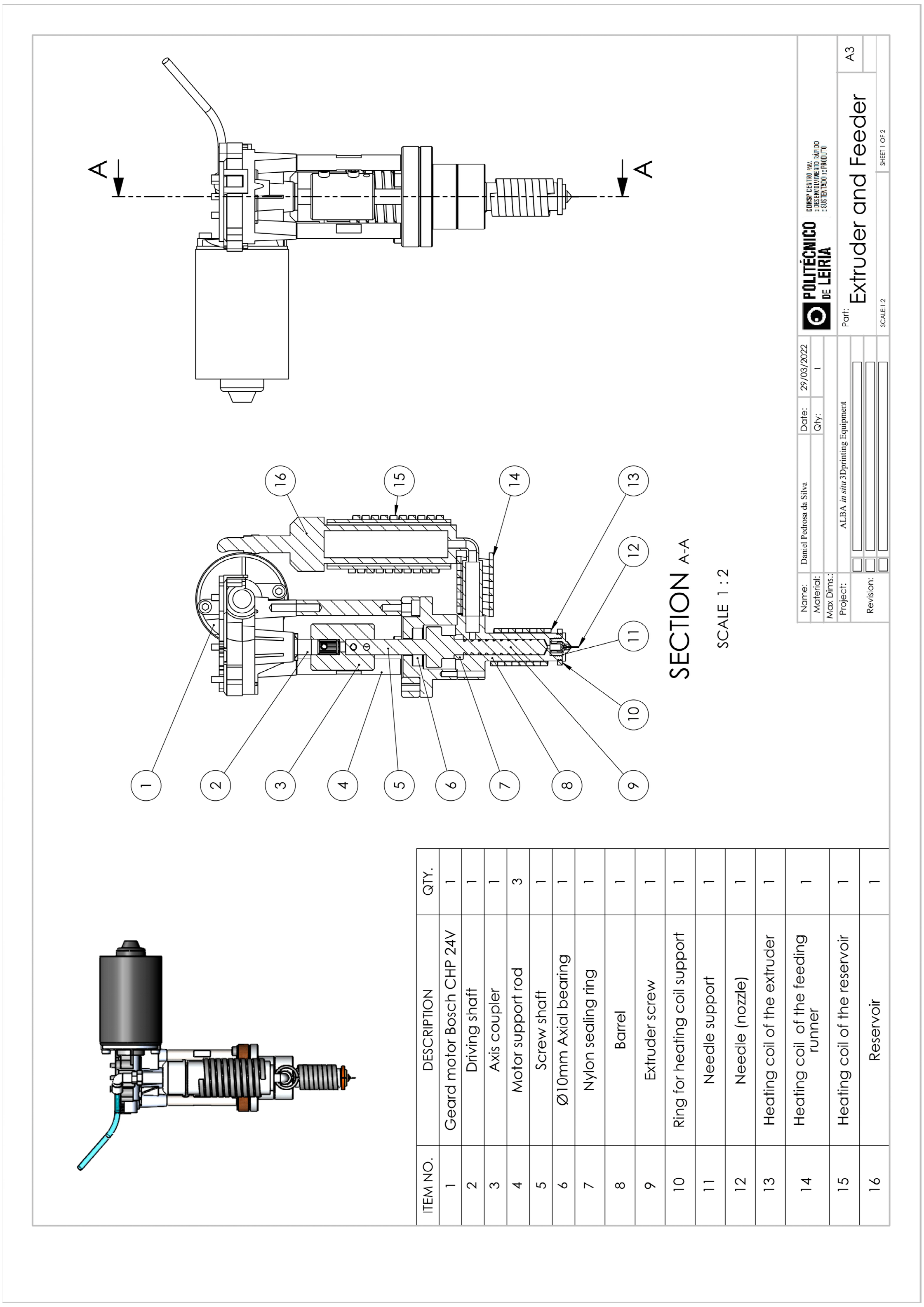

Figure S3—Detailed drawing of the extruder.

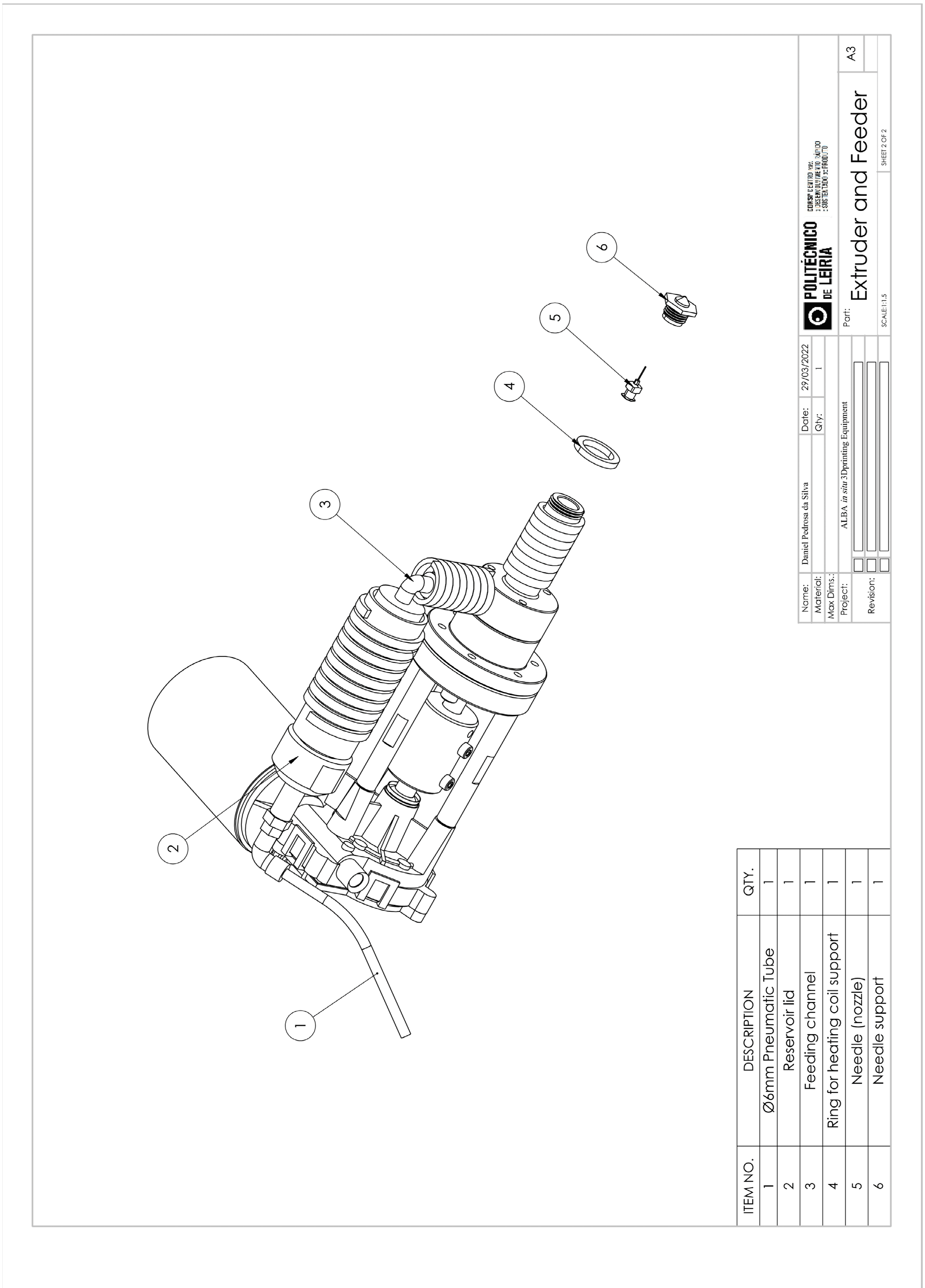

Figure S4—Representation of the extruder with the exploded view of the nozzle.
